# Supplementary material for: Impact of Amerind ancestry and FADS genetic variation on omega-3 deficiency and cardiometabolic traits in Hispanic populations
Source: Commun Biol. 2021 Jul 28;4:918. doi: 10.1038/s42003-021-02431-4 (PMC8319323; doi:10.1038/s42003-021-02431-4)
Supplement: Supplementary file 1 — Supplementary Information [file 42003_2021_2431_MOESM1_ESM.pdf]

Supplementary Information for

"Impact of Amerind ancestry and *FADS* genetic variation on omega-3 deficiency and cardiometabolic traits in Hispanic populations"

Chaojie Yang, Brian Hallmark, Jin Choul Chai, Timothy D. O'Connor, Lindsay M. Reynolds, Alexis C. Wood, Michael Seeds, Yii-Der Ida Chen, Lyn M. Steffen, Michael Y. Tsai, Robert C. Kaplan, Martha L. Daviglus, Lawrence J. Mandarino, Amanda M. Fretts, Rozenn N. Lemaitre, Dawn K. Coletta, Sarah A. Blomquist, Laurel M. Johnstone, Chandra Tontsch, Qibin Qi , Ingo Ruczinski, Stephen S. Rich, Rasika A. Mathias, Floyd H. Chilton, Ani Manichaikul

Supplementary Methods

Dietary fish intake and supplement use in MESA

At the baseline examination, usual dietary intake over the previous year was assessed with a modified Block-style 120-item Food Frequency Questionnaire (FFQ).[1,2] Fish intake was quantified as the sum of the servings per day for the following food categories: fried fish, shrimp, tuna, boiled fish, fish stew and stir-fried shrimp. Use of nutritional supplements including "Cod liver oil, other fish oils or omega-3 fatty acids" was also reported in the MESA FFQ; we used these responses to verify that none of the participants included in our analyses reported use of these supplements.

Additional phenotypes in MESA

Fasting blood samples were drawn, processed and stored using standardized procedures. Total cholesterol, HDL- cholesterol and triglycerides were measured at the Collaborative Studies Clinical Laboratory at Fairview-University Medical Center (Minneapolis, MN). Total cholesterol was measured using a cholesterol oxidase method (Roche Diagnostics; Indianapolis, IN), HDL-cholesterol using the cholesterol oxidase method (Roche Diagnostics) after precipitation of non-HDL-cholesterol with magnesium/dextran, triglycerides using Triglyceride GB reagent (Roche Diagnostics). All lipid measurements were performed on the Roche COBAS FARA centrifugal analyzer.[3]

Height was measured to the nearest 0.1 cm with the subject in stocking feet and weight was measured to the nearest pound with the subject in light clothing using a balanced scale. Waist circumference (WC), and hip circumference were measured to the nearest 0.5 kg, 0.1 cm, and 0.1 cm, respectively.

Plasma soluble ICAM-1 (sICAM-1), and soluble E-selectin (sE-selectin) concentrations were measured in blood samples collected at baseline and were processed with the use of a standardized protocol based on that used in the Cardiovascular Health Study[4] and stored at –80 °C until analyzed at the University of Vermont (Burlington, VT).[5]

Detection of outliers in measured PUFA levels and selected proximal traits

Outliers for all of the quantitative traits examined by genetic association analysis were identified by the limits of median +/- 3.5 * MAD', where MAD' is computed with a scale factor constant of 1.4826 [default for the mad() function in R]. The value of MAD' = 1.4826 * MAD_0 where MAD_0 is the raw value of median absolute deviation. For the all of PUFA traits, as well as the selected lipid levels, anthopometric traits, and inflammatory markers, outliers were winsorized to the value of (median +/- 3.5 * MAD').

Genetic association analysis

Within the n-3 and n-6 LC-PUFA study, outliers were identified using a cutoff based on the median absolute deviation (MAD) and winsorized the selected extreme values for each PUFA level **(Supplementary Figures 5-6).** In analysis of PUFA levels, we included covariate adjustment for age, sex, study site, principal components of ancestry, batch for fatty acid measurement and *FADS* cluster SNP rs174537. ProbABELv0.5.0 [6] was used to conduct genetic association analysis using linear models with robust standard errors. In addition, genetic association results were filtered based on minor allele frequency > 0.05 and imputation R squared > 0.5.

Local association analysis was performed for the 500 kb regions from starting position 61.3 Mb to ending position 61.8 Mb (for human genome build 37) on chromosome 11, and local association plots were generated using LocusZoom [7]. Statistical significance for local association analysis

was determined using a Bonferroni-corrected *P*-value with a family-wise error rate α=0.05, correcting for the total number 1081 of genetic variants passing filters in the region of interest. Thus, our genetic association analyses were subject to a Bonferroni corrected threshold of $P^*=0.05 / 1081=4.6\times10^{-5}$.

Global Ancestry Analysis

Individuals from all of the the1000 Genomes phase 3[8] and the Human Genome Diversity Project (HGDP)[9,10] groups listed below were included in global ancestry analysis in order to inform labels for the inferred ancestry groups.

1000 Genomes populations:

- Amerind: MXL (Mexican Ancestry from Los Angeles, USA), PUR (Puerto Ricans from Puerto Rico), CLM (Colombians from Medellin, Colombia) and PEL (Peruvians from Lima, Peru) were used to represent Amerind populations from the 1000 Genomes.
- African: ASW (Americans of African Ancestry in SW, USA), ACB (African Caribbeans in Barbados), ESN (Esan in Nigeria); GWD (Gambian in Western Divisions in the Gambia); LWK (Luhya in Webuye, Kenya), MSL (Mende in Sierra Leone) and YRI (Yoruba in Ibadan, Nigeria) groups were used to represent African populations from the 1000 Genomes.
- European: Utah Residents (CEPH) with Northern and Western European Ancestry (CEU), Toscani in Italia (TSI), Finnish in Finland (FIN), British in England and Scotland (GBR), Iberian Population in Spain (IBS) set were used to represent the non-admixed European population from the 1000 Genomes.

Human Genome Diversity Project (HGDP):

- Amerind: Colombian, Karaitiana, Maya, Pima and Surui were used to represent Amerind populations in the HGDP.

- African: Bantu, Biaka, Mandenka, Mbuti pygmy, Mozabite, San and Yoruba were used to represent African populations in the HGDP.
- European: Adygei, Basque, French, North Italian, Orcadian, Russian, Sardinian and Tuscan were used to represent the non-admixed European population in the HGDP.

Model-based cluster analysis was applied to estimate proportions of European, African and Amerind ancestry for all genotyped individuals using the package ADMIXTURE[11]. This software package used a block relaxation approach implemented with a novel quasi-Newton acceleration method that makes the method computationally feasible for much larger data sets, both in terms of the number of individuals and the number of genetic variants.

Local Ancestry Analysis

In order to construct an appropriate reference panel for local ancestry analysis, we sought to identify representative non-admixed Amerind, African and European individuals from among the 1000 Genomes phase 3[8] and the HGDP[9,10] groups included in global ancestry analysis, as noted above. As previous research has noted a high degree of admixture among some of the 1000 Genomes Amerind populations[12], we initially examined the mean global proportion of Amerind ancestry in each of the putative Amerind groups from the 1000 Genomes and the HGDP. Similar to the previous report[12], we noted the overall global proportion of Amerind ancestry was <0.5 in each of MXL, PUR and CLM from the 1000 Genomes. Thus, we removed these groups from further consideration. We confirmed that the remaining 1000 Genomes PEL group, as well as all of the HGDP Amerind groups exhibited mean global Amerind ancestry > 0.7. Thus, these groups were carried forward for further consideration. We further cleaned the remaining 1000 Genomes and HGDP reference samples for each of the continental super-populations, by applying a threshold for each population, such that inclusion in the Amerind, African or European reference panel used for local ancestry analysis required at least 90%, 99% or 99% global proportions of

ancestry for the respective continent represented. After applying this filter, there were 36, 371 and 434 individuals included in the resulting reference panels for Amerind, African and European ancestry, respectively.

Local ancestry was defined as the genetic ancestry of each individual for the region of *FADS* SNP rs174537, where each individual can have 0, 1 or 2 copies of an allele derived from each ancestral population. Local ancestry, was estimated using the RFMix package.[13] This tool implements a discriminative modeling approach by dividing the region on chromosome 11 into windows. Local ancestry is inferred within each window by using a conditional random field (CRF), parameterized by random forest trained using reference data representing the selected continental source populations (European, Amerind and African).

Follow-up in the AIR and HCHS/SOL cohorts

Arizona Insulin Resistance (AIR) registry: The AIR registry comprises 667 individuals (aged 8-83 years).[14] Within the AIR registry, there are 497 adult Hispanic participants (18-83 years) who agreed and consented to have their samples stored and analyzed for future projects (such as this study). Genotyping of *FADS* rs174537 was performed using a TaqMan SNP genotyping assay. Anthropometric data for the AIR registry participants include weight, height, body mass index, % body fat, hip circumference, and waist circumference. The cardio-metabolic measures obtained from each participant included glucose, hemoglobin A1C, insulin, triglycerides, total cholesterol, high-density lipoprotein cholesterol (HDL), low-density lipoprotein cholesterol (LDL) and very-low-density lipoprotein cholesterol (VLDL).

Hispanic Community Health Study (HCHS/SOL): The HCHS/SOL is a prospective population-based study of 16,415 Hispanic/Latino adults aged 18 to 74 years at recruitment who were living in four US metropolitan areas (Bronx, NY; Chicago, IL; Miami, FL; and San Diego, CA).[15,16] A comprehensive battery of interviews and a clinical assessment with blood draw (both fasting and two hours after a 75 gram glucose load) were conducted at in-person clinic visits during 2008 to 2011 (Visit 1 baseline) and during 2014 to 2017 (Visit 2). Information on demographics, behaviors, health status, family and medical histories, and medication use was collected using structured questionnaires, and blood pressure and anthropometric traits were measured. Blood biomarkers including blood lipids and glycemic traits, were measured by standard methods.[17] Annual follow-up telephone interviews were conducted to ascertain information on health status.

Genotyping was performed with an Illumina custom array (15041502 B3), which consists of the Illumina Omni 2.5M array (HumanOmni2.5-8v1-1) plus ~150k custom SNPs, with the quality control (QC) performed at HCHS/SOL Genetic Analysis Center. The variant rs174537 was genotyped directly through this effort in HCHS/SOL. An iterative procedure was used to simultaneously estimate principal components (PCs) reflecting population structure and kinship coefficients measuring familial relatedness.[18–20]

Supplementary Table 1: Regression of n-3 and n-6 PUFAs on global ancestry, also accounting for local ancestry and rs174537 SNP genotype.

Model 1								
	Columbia University		UCLA		University of Minnesota		Meta Analysis	
	BETA	P-value	BETA	P-value	BETA	P-value	BETA	P-value
EPA (%)	-0.22	0.097	-0.38	1.6×10^{-05}	-0.65	1.7×10^{-14}	-0.48	3.7×10^{-23}
DPA (%)	-0.14	0.086	-0.14	0.036	-0.27	1.4×10^{-04}	-0.18	7.6×10^{-06}
DHA (%)	-0.34	0.402	-0.33	0.325	-0.91	6.3×10^{-04}	-0.63	0.0007
ARA (%)	-4.86	9.8×10^{-07}	-4.08	3.2×10^{-07}	-3.45	4.6×10^{-05}	-4.06	1.3×10^{-16}
Model 2								
	Columbia University		UCLA		University of Minnesota		Meta Analysis	
	BETA	P-value	BETA	P-value	BETA	P-value	BETA	P-value
EPA (%)	-0.05	0.702	-0.16	0.067	-0.51	5.5×10^{-09}	-0.28	3.7×10^{-08}
DPA (%)	-0.01	0.873	0.02	0.792	-0.18	0.017	-0.05	0.185
DHA (%)	0.09	0.844	-0.05	0.883	-0.71	0.011	-0.37	0.056
ARA (%)	-1.47	0.084	-0.75	0.287	-0.64	0.372	-0.89	0.038
Model 3								
	Columbia University		UCLA		University of Minnesota		Meta Analysis	
	BETA	P-value	BETA	P-value	BETA	P-value	BETA	P-value
EPA (%)	0.08	0.570	-0.12	0.179	-0.48	3.1×10^{-08}	-0.34	8.4×10^{-07}
DPA (%)	0.04	0.683	0.03	0.653	-0.14	0.059	-0.07	0.192
DHA (%)	0.20	0.696	0.06	0.879	-0.69	0.018	-0.47	0.058
ARA (%)	-1.16	0.199	-0.39	0.583	-0.08	0.914	-0.52	0.364

Regression analysis results for Model 1-3 stratified by study site and combined by meta-analysis. All beta estimates for effects of the global ancestry on fatty acid levels are presented in units of percent total fatty acids. *P*-values are based on a Z-test from inverse variance weighted meta-analysis carried out with a total of n = 1057.

Model 1. PUFA ~ age + sex + fish intake + global proportions of Amerind ancestry
Model 2. PUFA ~ age + sex + fish intake + global proportions of Amerind ancestry + rs174537 genotype
Model 3. PUFA ~ age + sex + fish intake + global proportions of Amerind ancestry + rs174537 genotype + local proportion of Amerind ancestry

Supplementary Table 2: Interaction of rs174537 with global and local ancestry

	Model 1							
	Columbia University		UCLA		University of Minnesota		Meta Analysis	
	BETA	P-value	BETA	P-value	BETA	P-value	BETA	P-value
EPA (%)	-0.29	0.154	0.05	0.643	0.15	0.184	0.05	0.496
DPA (%)	-0.04	0.778	-0.04	0.672	-0.04	0.662	-0.04	0.503
DHA (%)	0.33	0.649	-0.03	0.958	0.02	0.948	0.05	0.852
ARA (%)	-0.09	0.939	0.46	0.624	-1.71	0.075	-0.49	0.4001
	Model 2							
	Columbia University		UCLA		University of Minnesota		Meta Analysis	
	BETA	P-value	BETA	P-value	BETA	P-value	BETA	P-value
EPA (%)	-0.06	0.265	-0.02	0.328	0.01	0.769	-0.02	0.366
DPA (%)	-0.003	0.927	-0.03	0.165	-0.05	0.118	-0.03	0.06
DHA (%)	0.17	0.417	0.13	0.289	0.06	0.587	0.105	0.177
ARA (%)	0.13	0.720	-0.18	0.457	-0.39	0.194	-0.178	0.279

Regression analysis results for interaction of rs174537 with global (Model 1) and local (Model 2) ancestry stratified by study site and combined by meta-analysis. All beta estimates for interaction effects on fatty acid levels are presented in units of percent total fatty acids. *P*-values are based on Z-tests from inverse variance weighted meta-analysis carried out with a total of n = 1057.

Model 1. PUFA ~ age + sex + fish intake + global proportions of Amerind ancestry + rs174537 genotype + local proportion of Amerind ancestry + rs174537 genotype * global proportion of Amerind ancestry
Model 2. PUFA ~ age + sex + fish intake + global proportions of Amerind ancestry + rs174537 genotype + local proportion of Amerind ancestry + rs174537 genotype * local proportion of Amerind ancestry

Supplementary Table 3: Genotypic effects of rs174537 on fasting lipids, anthropometric and inflammatory traits.

			Model 1		Model 2	
			Beta	P value	Beta	P value
Fasting Lipids	Triglycerides (mg/dL)	GT	21.27	0.0001	11.81	0.044
		TT	29.94	1.01×10^{-06}	16.19	0.018
	HDL-C (mg/dL)	GT	-1.30	0.141	-0.35	0.708
		TT	-2.48	0.010	-0.95	0.384
Anthropometrics	waist-hip ratio	GT	0.006	0.152	-0.002	0.710
		TT	0.013	8.94×10^{-03}	-0.0007	0.908
	Height (cm)	GT	-1.36	0.002	-0.39	0.412
		TT	-3.46	6.59×10^{-12}	-1.62	0.003
	Weight (kg)	GT	-1.89	0.077	-1.36	0.230
		TT	-3.12	7.48×10^{-03}	-2.41	0.067
	BMI (kg/m^2)	GT	-0.25	0.50	-0.39	0.33
		TT	-0.002	0.99	-0.37	0.42
Inflammatory	s-ICAM (ng/mL)	GT	30.64	0.002	19.09	0.081
		TT	26.09	0.018	10.22	0.411
	E-Selectin (ng/mL)	GT	10.00	0.048	10.41	0.051
		TT	11.50	0.032	10.49	0.080

Regression analysis results for the effect of rs174537 genotype compared to two copies of the reference allele G on inflammatory, fasting Lipids and anthropometrics with adjustment for age and sex (Model 1) and Model 1 + first four principal components of ancestry (Model 2). The sample sizes are 1102 (GG: 293; GT: 484; TT: 325) for waist-hip ratio; height; weight and BMI, 1101 (GG: 293 ;GT: 483 ;TT: 325) for triglycerides and HDL-C, 439 (GG: 112; GT: 194; TT: 133) for s-ICAM, and 183 (GG: 48; GT: 76; TT: 59) for E-Selectin. *P*-values are calculated using two-sided t-tests for each regression coefficient.

Supplementary Table 4: Genotypic effects of rs174557 on Fasting Lipids, Anthropometrics and Inflammatory Traits.

			Model 1		Model 2	
			Beta	P value	Beta	P value
Fasting Lipids	Triglycerides (mg/dL)	AG	22.92	2.55×10^{-05}	14.21	0.012
		GG	29.94	9.56×10^{-07}	16.99	0.012
	HDL-C (mg/dL)	AG	-1.48	0.085	-0.59	0.513
		GG	-2.40	0.012	-0.93	0.390
Anthropometrics	waist-hip ratio	AG	0.008	0.095	-0.0004	0.932
		GG	0.013	0.008	-0.0002	0.972
	Height (cm)	AG	-1.66	0.0001	-0.747	0.103
		GG	-3.84	1.57×10^{-14}	-2.079	1.48×10^{-04}
	Weight (kg)	AG	-4.18	0.068	-2.95	0.219
		GG	-7.46	0.003	-6.04	0.036
	BMI (kg/m^2)	AG	-0.14	0.64	-0.25	0.522
		GG	0.05	0.91	-0.32	0.483
Inflammatory	s-ICAM (ng/mL)	AG	30.24	0.002	20.02	0.058
		GG	26.14	0.017	11.54	0.343
	E-Selectin (ng/mL)	AG	7.29	0.139	7.21	0.161
		GG	8.83	0.101	6.93	0.239

Regression analysis results for the effect of rs174557 genotype compared to two copies of the reference allele A on inflammatory, fasting Lipids and anthropometrics with adjustment for age and sex (Model 1) and Model 1 + first four principal components of ancestry (Model 2). The sample sizes are 1102 (GG: 293; GT: 484; TT: 325) for waist-hip ratio; height, weight and BMI;, 1101 (GG: 293 ;GT: 483 ;TT: 325) for triglycerides and HDL-C, 439 (GG: 112; GT: 194; TT: 133) for s-ICAM, and 183 (GG: 48; GT: 76; TT: 59) for E-Selectin. *P*-values are presented based on two-sided t-tests for each regression coefficient.

Supplementary Table 5. Participant characteristics for the AIR registry.

Characteristics	N = 497
Sex (Female)	64.60%
Age (Years)	36.4 (11.2)
Height (cm)	163 (8.70)
Weight (kg)	80.8 (19.5)
HDL-C (mg/dL)	43.9 (11.2)
LDL-C (mg/dL)	106.6(28.6)
Triglycerides (mg/dL)	136.6 (79.7)
Total Cholesterol (mg/dL)	173.9 (34.9)
Body mass index (kg/m^2)	29.9 (5.8)
Waist-to-Hip ratio	0.9 (0.1)

Phenotypic descriptive statistics are presented as percentages for dichotomous variables and mean (standard deviation) for continuous variables.

Supplementary Table 6. Participant characteristics for the HCHS/SOL cohort.

Characteristics	Self-reported Hispanic country/region of origin						Total (N=12333)
	**Cuba** (N=2190)	**Dominican** (N=1157)	**PuertoRico** (N=2178)	**SouthAmer** (N=899)	**CentralAmer** (N=1335)	**Mexico** (N=4537)	
Sex (Female)	(53.8%)	(65.7%)	(58.3%)	(60.2%)	(60.1%)	(60.9%)	(59.5%)
Age (years)	48.7 (13.3)	45.5 (14.3)	47.7 (14.3)	46.4 (13.3)	44.5 (13.4)	44.6 (13.9)	46.1 (13.9)
Height (cm)	164.1 (9.2)	162 (8.9)	163 (9.4)	160.8 (9.3)	160 (8.7)	161.3 (9.4)	162.0 (9.3)
Weight (kg)	78.9 (17.7)	77.4 (16.5)	82 (20.1)	74 (15.5)	76.9 (16.6)	77.8 (17.8)	78.4 (17.9)
BMI (kg/m^2)	29.3 (5.9)	29.5 (5.8)	30.9 (7)	28.6 (5.2)	30 (5.8)	29.9 (6)	29.8 (6.1)
Waist-hip ratio	0.9 (0.1)	0.9 (0.1)	0.9 (0.1)	0.9 (0.1)	0.9 (0.1)	0.9 (0.1)	0.9 (0.1)
HDL (mg/dl)	49 (12.9)	51.6 (13)	49.5 (13.5)	50.4 (13.2)	48.6 (12.9)	49.2 (12.6)	49.5 (13.0)
LDL (mg/dl)	130.1 (38)	121.3 (38.8)	115.9 (35.6)	126.1 (35.5)	124 (35.8)	122.2 (35.4)	122.9 (36.6)
Triglycerides (mg/dl)	134.6 (71.2)	107.3 (57.7)	122.6 (66.4)	131.7 (71.5)	142.6 (72.7)	132.9 (66.9)	129.8 (68.4)
Total cholesterol (mg/dl)	206 (44)	194.4 (43.2)	189.8 (41.5)	203 (41.6)	201.1 (41.7)	198 (41.1)	198.3 (42.3)
C-Reactive orotein (CRP) (mg/L)	4.3 (6.6)	3.9 (6.6)	4.7 (6.9)	3.4 (7.7)	4.3 (10.3)	3.8 (7.3)	4.1 (7.5)
Effect Allele Frequency of rs174537 (effect allele: T)	0.28	0.26	0.38	0.63	0.61	0.63	0.48

Rhenotypic descriptive statistics are presented as percentages for dichotomous variables and mean (standard deviation) for continuous variables.

Supplementary Table 7. Analysis of rs174537 effects on triglycerides and waist-hip ratio in AIR registry and HCHS/SOL.

	**AIR cohort**		**HCHS/SOL cohort**	
	Beta	P-value	Beta	P-value
Triglycerides	10.39	0.03	8.75	5.84×10^{-25}
Waist-hip ratio	0.01	0.20	0.003	2.77×10^{-05}
Height	-0.49	0.20	-1.33	4.47×10^{-56}
Weight	0.43	0.71	-1.25	2.61×10^{-08}

Genetic additive effects of *FADS* rs174537 allele T on triglycerides, waist-hip ratio, height and weight in the AIR registry and the HCHS/SOL cohort. The effect estimates are adjusted for age and sex in both studies (plus additional random-effects adjustment for household block and unit sharing in HCHS/SOL). *P*-values are calculated using two-sided t-tests for each regression coefficient derived with n = 497 in AIR and n = 12,333 in HCHS/SOL.

Supplementary Table 8: Stratified analysis of rs174537 effects on triglycerides and waist-hip ratio HCHS/SOL, with additional adjustment for principal components of ancestry.

	Chicago		Miami		San Diego		Bronx		Meta Analysis	
	BETA	P-value	BETA	P-value	BETA	P-value	BETA	P-value	BETA	P-value
Triglycerides	4.94	0.015	2.85	0.13	4.47	0.014	4.04	0.019	4.05	1.26×10^{-05}
Waist-hip ratio	-0.0005	0.79	-0.003	0.096	-0.0008	0.63	-0.003	0.19	-0.002	0.068
Height	0.006	0.975	-0.035	0.834	-0.163	0.354	-0.33	0.06	-0.129	0.138
Weight	-0.034	0.943	0.293	0.518	0.465	0.369	-1.02	0.038	-0.068	0.778

Genetic additive effects of *FADS* SNP rs174537 allele T on triglycerides, waist-hip ratio, height and weight in the HCHS/SOL cohort followed by a fixed-effects meta-analysis. The effect estimates are adjusted for age, sex, Hispanic region of origin, first 5 principal components of ancestry, household block and unit sharing. *P*-values were derived from Z-tests for inverse variance weighted meta-analysis with a total sample size of n = 12,333.

Supplementary Table 9: Regression of n-3 and n-6 PUFAs on Study Site in MESA Hispanic Participants

		BETA	P-value
EPA (% of total LC-PUFA)	UCLA	-0.22	3.81×10^{-20}
	University of Minnesota	-0.19	8.72×10^{-21}
DPA (% of total LC-PUFA)	UCLA	-0.06	0.0003
	University of Minnesota	-0.08	3.10×10^{-09}
DHA (% of total LC-PUFA)	UCLA	-1.34	6.36×10^{-54}
	University of Minnesota	-1.02	2.25×10^{-41}
ARA (% of total LC-PUFA)	UCLA	-1.55	2.39×10^{-15}
	University of Minnesota	-1.37	2.60×10^{-15}

Study-site effects on LC-PUFA levels are reported based on assignment of Columbia University as the reference group. Results are based on covariate adjustment for age and sex. *P*-values are calculated using two-sided t-tests for each regression coefficient.

Supplementary Figure 1: Relationship of LC-PUFA levels with Global Proportion of Amerind Ancestry before and after adjustment for rs174557 genotype.

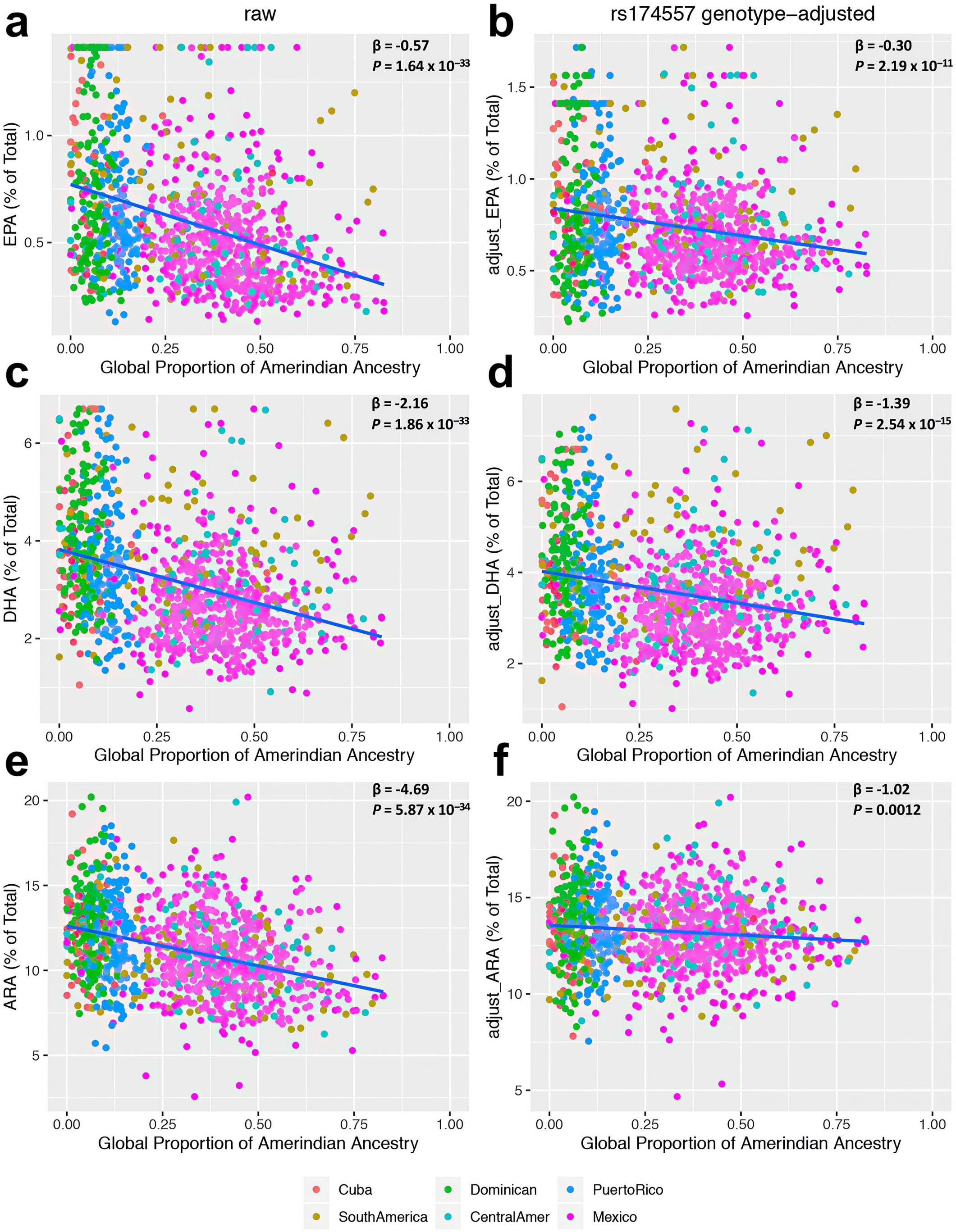

The regression effect estimates (β expressed as % of total fatty acids) and *P*-values are shown in the upper right corner of each panel. The relationship of LC-PUFA levels with Global Proportion of Amerind Ancestry as estimated from genome-wide SNP data is shown for (a) EPA - raw, (b) EPA – genotype-adjusted, (c) DHA – raw, (d) DHA – genotyped-adjusted, (e) ARA – raw, and (f) ARA – genotype-adjusted. Here, the rs174557 genotype-adjusted LC-PUFA levels were obtained as residuals after regression against rs174557 genotype and re-centered around the raw mean. *P*-values are presented based on two-sided t-tests for each regression coefficient derived with n = 1102 biologically independent samples.

Supplementary Figure 2: Relationship of triglycerides with Global Proportion of Amerind Ancestry before and after adjustment for rs174557 genotype.

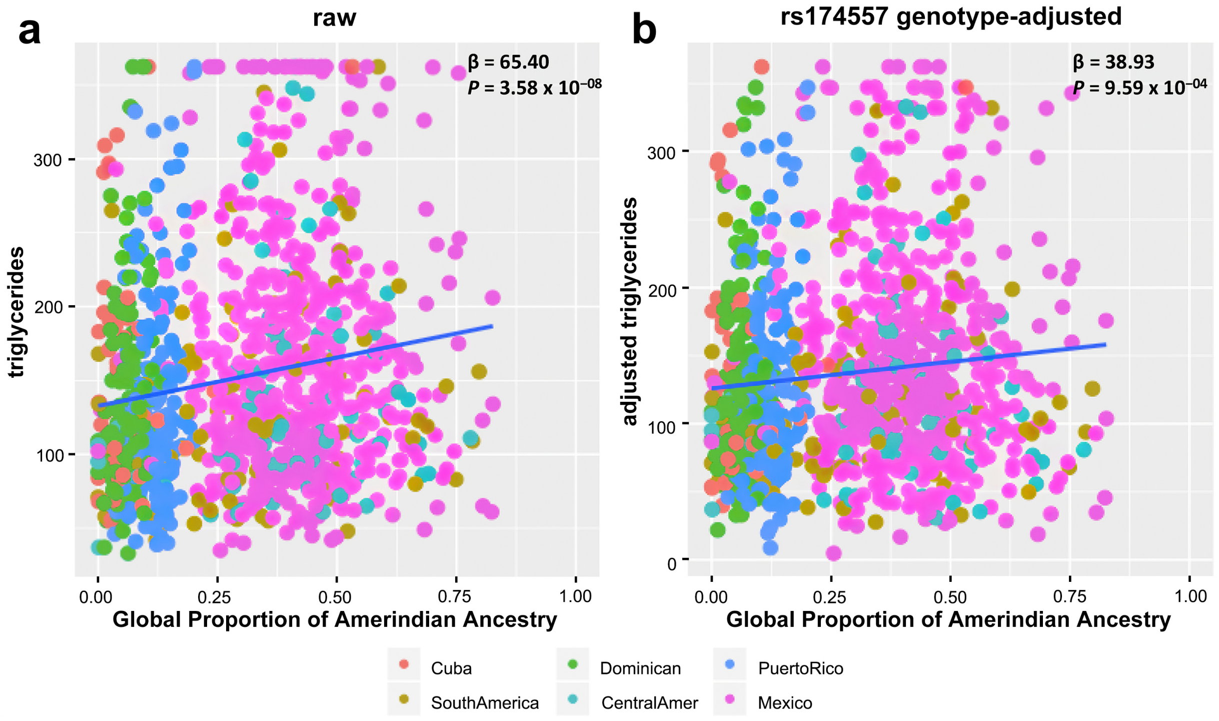

The regression effect estimates (β in mg/dL) and *P*-values are shown in the upper right corner of each panel. The relationships are shown for each of (a) raw triglyceride levels, and (b) genotype-adjusted tryglyceride levels with Global Proportion of Amerind Ancestry. Here, rs174557 genotype-adjusted triglyceride levels were obtained as residuals from regression accounting for rs174557 genotype, and re-centered around the raw means. *P*-values are presented based on two-sided t-tests for each regression coefficient derived with n = 1101 biologically independent samples.

Supplementary Figure 3: Conditional local association plots for n-3 and n-6 PUFAs in the *FADS1/2* region, accounting for the rs174537 SNP.

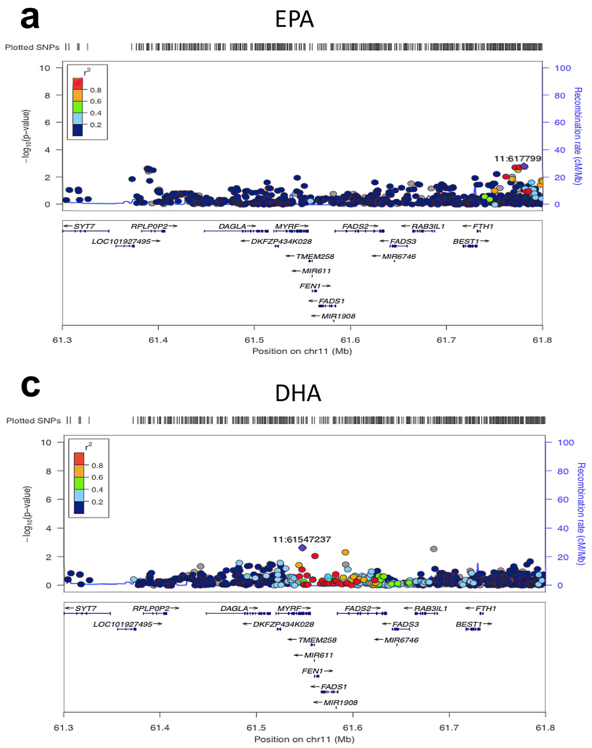

The plots present -log10 p-values for the association of each genetic variant within the region shown with the fatty acid traits. *P*-values were derived based on two-sided t-tests for the genetic additive effects with n = 1,102 biologically independent samples. Genetic coordinates on the x-axis are denoted based on human genome Build 37.

Supplementary Figure 4: Genotypic effects of rs174537 on triglycerides and E-selectin.

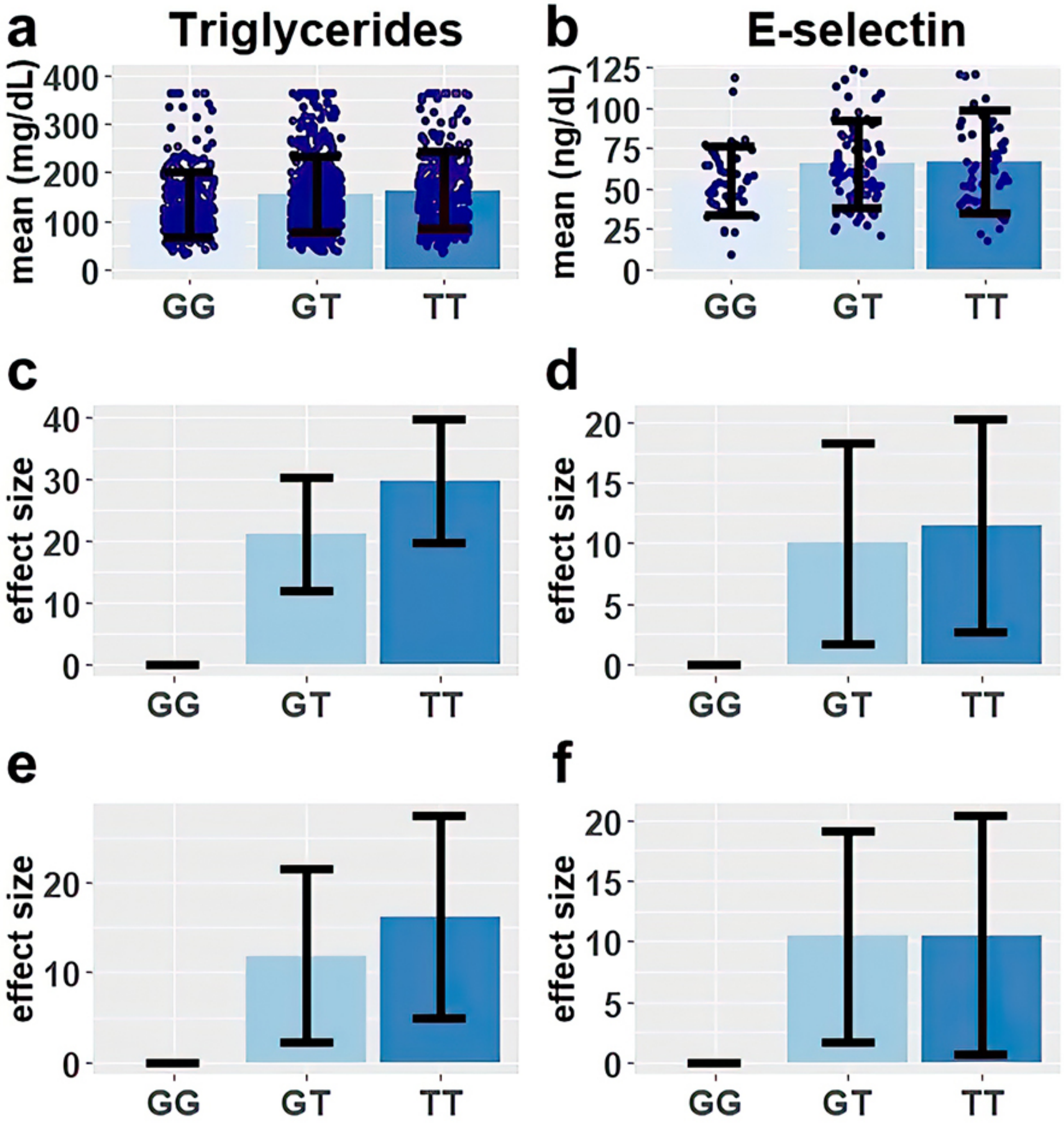

Mean and standard deviation of (a) triglycerides, and (b) E-selectin stratified by the genotypes of rs174537. Estimated effect and standard error among participants carrying one or two copies of the ancestral allele T (compared to the reference of zero) for (c) triglycerides, and (d) E-selectin after adjustment for age and sex. Estimated effect and standard error among participants carrying one or two copies of the ancestral allele T (compared to a reference of zero) for (e) triglycerides; and (f) E-selectin, after adjustment for age, sex and principal components of ancestry. The sample sizes are 1101 (GG: 293 ;GT: 483 ;TT: 325) for triglycerides and 183 (GG: 48; GT: 76; TT: 59) for E-Selectin. Source data are provided in Supplementary Data 1.

Supplementary Figure 5: Raw distribution of n-3 and n-6 PUFAs in MESA Hispanic participants.

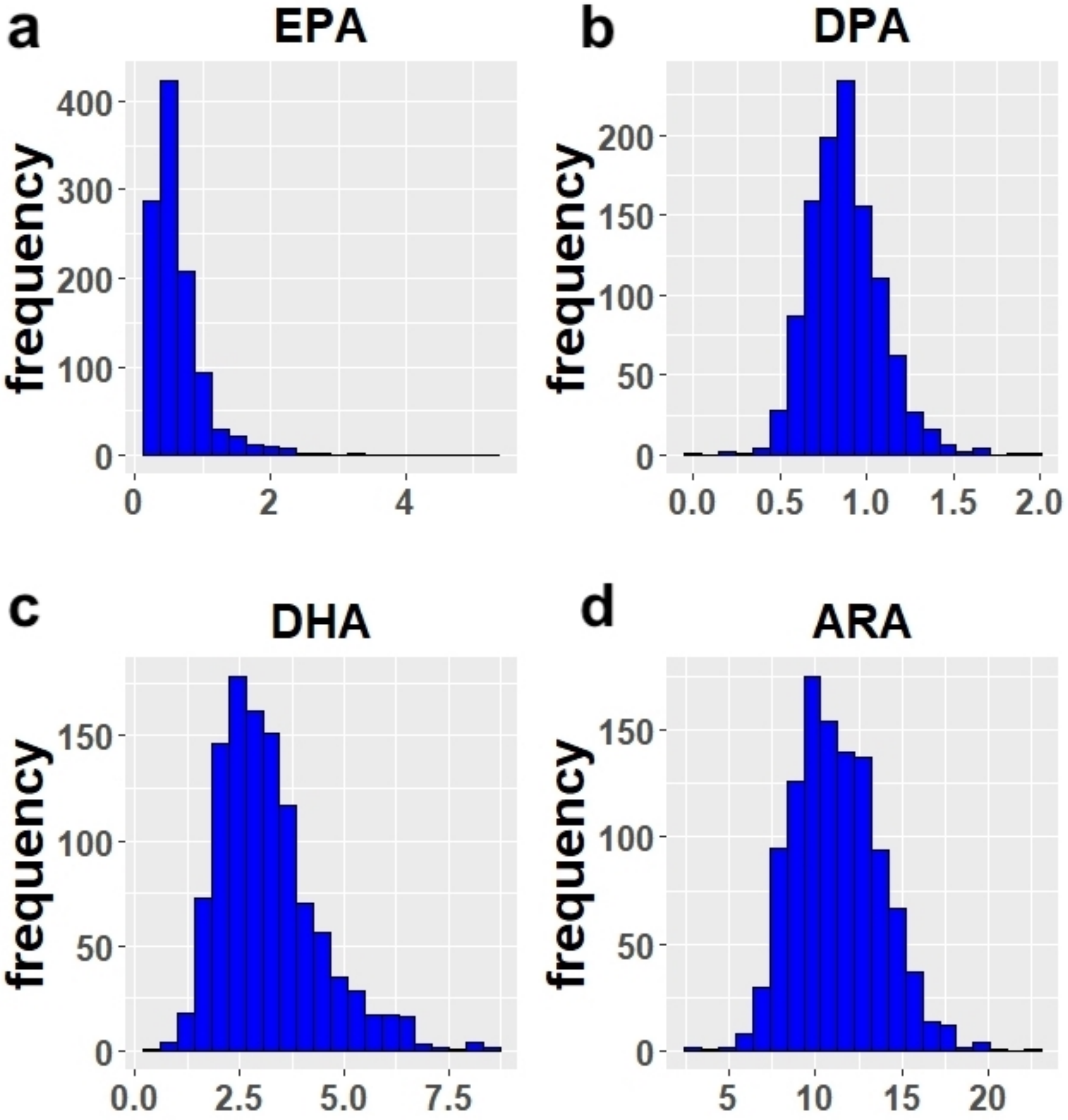

Values for (a) EPA, (b) DPA, (c) DHA, and (d) ARA are presented in units of % of total fatty acids. Source data for the histograms are presented in Supplementary Data 2.

Supplementary Figure 6: Distribution of n-3 and n-6 PUFAs in MESA Hispanic participants after winsorizing at median +/- 3.5 Median Absolute Deviation (MAD).

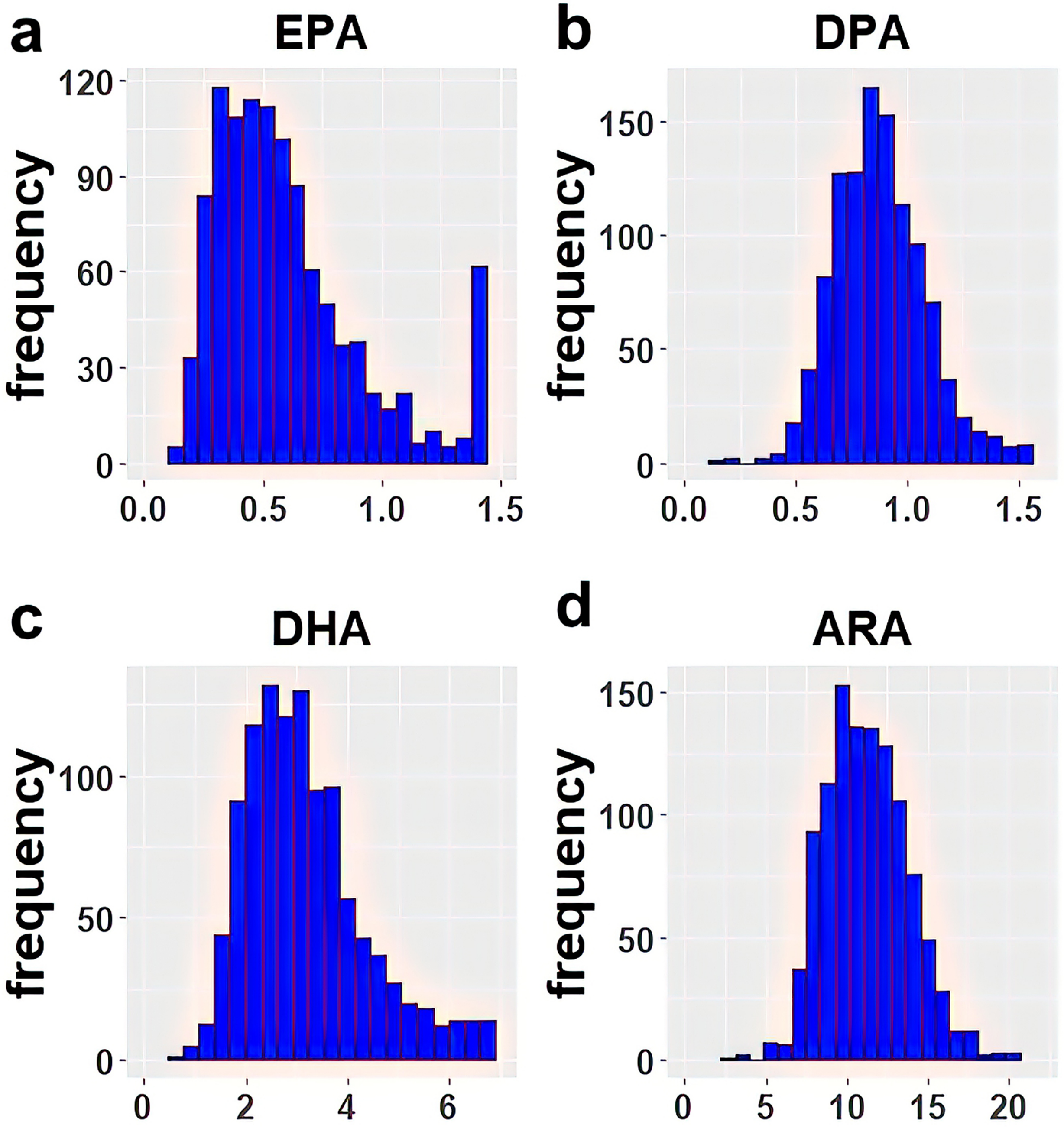

Values for (a) EPA, (b) DPA, (c) DHA, and (d) ARA are presented in units of % of total fatty acids. Source data for the histograms are provided in Supplementary Data 3.

Supplementary References

1. Mayer-Davis, E. J. *et al.* Validity and reproducibility of a food frequency interview in a Multi-Cultural Epidemiology Study. *Ann. Epidemiol.* **9**, 314–324 (1999).
2. Nettleton, J. A., Rock, C. L., Wang, Y., Jenny, N. S. & Jacobs, D. R. Associations between dietary macronutrient intake and plasma lipids demonstrate criterion performance of the Multi-Ethnic Study of Atherosclerosis (MESA) food-frequency questionnaire. *Br. J. Nutr.* **102**, 1220–1227 (2009).
3. Holvoet, P. *et al.* The relationship between oxidized LDL and other cardiovascular risk factors and subclinical CVD in different ethnic groups: the Multi-Ethnic Study of Atherosclerosis (MESA). *Atherosclerosis* **194**, 245–252 (2007).
4. Cushman, M., Cornell, E. S., Howard, P. R., Bovill, E. G. & Tracy, R. P. Laboratory methods and quality assurance in the Cardiovascular Health Study. *Clin. Chem.* **41**, 264–270 (1995).
5. Arnett, D. K. *et al.* Biomarkers of inflammation and hemostasis associated with left ventricular mass: The Multiethnic Study of Atherosclerosis (MESA). *Int. J. Mol. Epidemiol. Genet.* **2**, 391–400 (2011).
6. Aulchenko, Y. S., Struchalin, M. V. & van Duijn, C. M. ProbABEL package for genome-wide association analysis of imputed data. *BMC Bioinformatics* **11**, 134 (2010).
7. Pruim, R. J. *et al.* LocusZoom: regional visualization of genome-wide association scan results. *Bioinforma. Oxf. Engl.* **26**, 2336–2337 (2010).
8. 1000 Genomes Project Consortium *et al.* A global reference for human genetic variation. *Nature* **526**, 68–74 (2015).
9. Li, J. Z. *et al.* Worldwide Human Relationships Inferred from Genome-Wide Patterns of Variation. *Science* **319**, 1100–1104 (2008).
10. Bergström, A. *et al.* Insights into human genetic variation and population history from 929 diverse genomes. *Science* **367**, (2020).

11. Alexander, D. H., Novembre, J. & Lange, K. Fast model-based estimation of ancestry in unrelated individuals. *Genome Res.* **19**, 1655–1664 (2009).

12. Harris, D. N. *et al.* Evolution of Hominin Polyunsaturated Fatty Acid Metabolism: From Africa to the New World. *Genome Biol. Evol.* **11**, 1417–1430 (2019).

13. Maples, B. K., Gravel, S., Kenny, E. E. & Bustamante, C. D. RFMix: A discriminative modeling approach for rapid and robust local-ancestry inference. *Am. J. Hum. Genet.* (2013) doi:10.1016/j.ajhg.2013.06.020.

14. Shaibi, G. Q., Coletta, D. K., Vital, V. & Mandarino, L. J. The design and conduct of a community-based registry and biorepository: a focus on cardiometabolic health in Latinos. *Clin. Transl. Sci.* **6**, 429–434 (2013).

15. Lavange, L. M. *et al.* Sample design and cohort selection in the Hispanic Community Health Study/Study of Latinos. *Ann. Epidemiol.* **20**, 642–649 (2010).

16. Sorlie, P. D. *et al.* Design and implementation of the Hispanic Community Health Study/Study of Latinos. *Ann. Epidemiol.* **20**, 629–641 (2010).

17. Qi, Q. *et al.* Objectively Measured Sedentary Time and Cardiometabolic Biomarkers in US Hispanic/Latino Adults: The Hispanic Community Health Study/Study of Latinos (HCHS/SOL). *Circulation* **132**, 1560–1569 (2015).

18. Conomos, M. P. *et al.* Genetic Diversity and Association Studies in US Hispanic/Latino Populations: Applications in the Hispanic Community Health Study/Study of Latinos. *Am. J. Hum. Genet.* **98**, 165–184 (2016).

19. Conomos, M. P., Miller, M. & Thornton, T. Robust Inference of Population Structure for Ancestry Prediction and Correction of Stratification in the Presence of Relatedness. *Genet. Epidemiol.* **39**, 276–293 (2015).

20. Conomos, M. P., Reiner, A. P., Weir, B. S. & Thornton, T. A. Model-free Estimation of Recent Genetic Relatedness. *Am. J. Hum. Genet.* **98**, 127–148 (2016).
