# Supplementary material for: Impact of Amerind ancestry and FADS genetic variation on omega-3 deficiency and cardiometabolic traits in Hispanic populations
Source: Commun Biol. 2021 Jul 28;4:918. doi: 10.1038/s42003-021-02431-4 (PMC8319323; doi:10.1038/s42003-021-02431-4)
Supplement: Supplementary file 5 — Reporting Summary [file 42003_2021_2431_MOESM5_ESM.pdf]

Corresponding author(s): Ani Manichaikul

Last updated by author(s): Jun 9, 2021

Reporting Summary

Nature Research wishes to improve the reproducibility of the work that we publish. This form provides structure for consistency and transparency in reporting. For further information on Nature Research policies, see our Editorial Policies and the Editorial Policy Checklist.

Statistics

For all statistical analyses, confirm that the following items are present in the figure legend, table legend, main text, or Methods section.

n/a	Confirmed	
☐	☒	The exact sample size (*n*) for each experimental group/condition, given as a discrete number and unit of measurement
☐	☒	A statement on whether measurements were taken from distinct samples or whether the same sample was measured repeatedly
☐	☒	The statistical test(s) used AND whether they are one- or two-sided<br>*Only common tests should be described solely by name; describe more complex techniques in the Methods section.*
☐	☒	A description of all covariates tested
☐	☒	A description of any assumptions or corrections, such as tests of normality and adjustment for multiple comparisons
☐	☒	A full description of the statistical parameters including central tendency (e.g. means) or other basic estimates (e.g. regression coefficient) AND variation (e.g. standard deviation) or associated estimates of uncertainty (e.g. confidence intervals)
☐	☒	For null hypothesis testing, the test statistic (e.g. *F*, *t*, *r*) with confidence intervals, effect sizes, degrees of freedom and *P* value noted<br>*Give P values as exact values whenever suitable.*
☒	☐	For Bayesian analysis, information on the choice of priors and Markov chain Monte Carlo settings
☒	☐	For hierarchical and complex designs, identification of the appropriate level for tests and full reporting of outcomes
☐	☒	Estimates of effect sizes (e.g. Cohen's *d*, Pearson's *r*), indicating how they were calculated

Our web collection on statistics for biologists contains articles on many of the points above.

Software and code

Policy information about availability of computer code

Data collection	We did not use custumized code for data collection.
Data analysis	- Genotype imputation in MESA was performed using the 1,000 Genomes Phase 3 integrated variant set completed using the Michigan Imputation Server (https://imputationserver.sph.umich.edu).<br>- We used ProbABELv0.5.0 to conduct genetic association analysis using linear models with robust standard errors.<br>- Local association plots were generated using LocusZoom.<br>- We estimated global proportions of European, African and Amerind ancestry using model-based cluster analysis implemented using the package ADMIXTURE.<br>- Local ancestry, was estimated using the RFMix package.<br>- Linear regression analyses will carried out in R.

For manuscripts utilizing custom algorithms or software that are central to the research but not yet described in published literature, software must be made available to editors and reviewers. We strongly encourage code deposition in a community repository (e.g. GitHub). See the Nature Research guidelines for submitting code & software for further information.

Data

Policy information about availability of data

All manuscripts must include a data availability statement. This statement should provide the following information, where applicable:
- Accession codes, unique identifiers, or web links for publicly available datasets
- A list of figures that have associated raw data
- A description of any restrictions on data availability

Individual level genotype data for MESA and HCHS/SoL are available through dbGaP. The dbGaP accession numbers are: Multi-Ethnic Study of Atherosclerosis

(MESA) phs001416, and the Hispanic Community Health Study / Study of Latinos (HCHS/SoL) phs001395.

Field-specific reporting

Please select the one below that is the best fit for your research. If you are not sure, read the appropriate sections before making your selection.

☒ Life sciences ☐ Behavioural & social sciences ☐ Ecological, evolutionary & environmental sciences

For a reference copy of the document with all sections, see nature.com/documents/nr-reporting-summary-flat.pdf

Life sciences study design

All studies must disclose on these points even when the disclosure is negative.

Sample size	The MESA participants included in our primary analysis comprised 1,102 unrelated Hispanic participants from the Multi-Ethnic Study of Atherosclerosis.
Data exclusions	Our analyses excluded outliers identified for the quantitative traits of interest. Outliers for all of the quantitative traits examined by genetic association analysis were identified by the limits of median +/- 3.5 * MAD', where MAD' is computed with a scale factor constant of 1.4826 [default for the mad() function in R].
Replication	We carried out replication of findings in MESA in two independent cohorts: the Arizona Insulin Resistance (AIR) registry and the Hispanic Community Health Study (HCHS/SOL).<br><br>The AIR registry comprises 667 individuals (aged 8-83 years) and has been described in detail elsewhere. Within the AIR registry, there are 497 adult Hispanic participants (18-83 years) who agreed and consented to have their samples stored and analyzed for future projects (such as this study).<br><br>The HCHS/SOL is a prospective population-based study of 16,415 Hispanic/Latino adults aged 18 to 74 years at recruitment who were living in four US metropolitan areas (Bronx, NY; Chicago, IL; Miami, FL; and San Diego, CA). Analyses for the current replication effort were restricted to those participants with genotype data and at least one of the phenotypes of interest available.
Randomization	There was no randomization as our project examined data from observational studies.
Blinding	There was no blinding because our data sets are derived from observational studies.

Reporting for specific materials, systems and methods

We require information from authors about some types of materials, experimental systems and methods used in many studies. Here, indicate whether each material, system or method listed is relevant to your study. If you are not sure if a list item applies to your research, read the appropriate section before selecting a response.

Materials & experimental systems

n/a	Involved in the study	
☒	☐	Antibodies
☒	☐	Eukaryotic cell lines
☒	☐	Palaeontology and archaeology
☒	☐	Animals and other organisms
☐	☒	Human research participants
☒	☐	Clinical data
☒	☐	Dual use research of concern

Methods

n/a	Involved in the study	
☒	☐	ChIP-seq
☒	☐	Flow cytometry
☒	☐	MRI-based neuroimaging

Human research participants

Policy information about studies involving human research participants

Population characteristics	Participants in the Multi-Ethnic Study of Atherosclerosis represent a cohort of individualsaged 45 to 84 years at baseline. For the current study, we focused on a subset of participants of self-reported Hispanic race/ethnicity with country-specific classification based on the birthplace of parents and grandparents corresponding to Central American (n=80), Cuban (n=45), Dominican (n=145), Mexican (n=572), Puerto Rican (n=167) and South American (n=93).
Recruitment	MESA Hispanic participants were recruited primarily from three field centers in the United States (Columbia University, University of California – Los Angeles (UCLA) and the University of Minnesota). Those with prevalent cardiovascular disease (CVD) were excluded to optimize the study of subclinical CVD progression and predictors of clinical CVD.
Ethics oversight	MESA participants were consented for participation at their respective MESA study sites, and the MESA study was also reviewed and approved by the Institutional Review Boards (IRBs) at each of the participating study sites. The current investigation including activities for analysis of LC-PUFA levels in MESA was reviewed and approved by the Institutional Review Board (IRB) at the University of Virginia.

Note that full information on the approval of the study protocol must also be provided in the manuscript.
