# Supplementary material for: Impact of Amerind ancestry and FADS genetic variation on omega-3 deficiency and cardiometabolic traits in Hispanic populations
Source: Commun Biol. 2021 Jul 28;4:918. doi: 10.1038/s42003-021-02431-4 (PMC8319323; doi:10.1038/s42003-021-02431-4)
Supplement: Supplementary file 7 — Description of Supplementary Files [file 42003_2021_2431_MOESM7_ESM.pdf]

Description of Additional Supplementary Files

File name: Supplementary Data 1

Description: Source data for Figure 3 and Supplementary Figure 4.

File name: Supplementary Data

Description: Source data for Supplementary Figure 5.

File name: Supplementary Data 3

Description: Source data for Supplementary Figure 6.
